# *IL2*-*IL21* gene cluster polymorphism is not associated with allograft function after kidney transplantation

**DOI:** 10.1007/s11255-014-0867-y

**Published:** 2014-11-08

**Authors:** Ewa Kwiatkowska, Leszek Domanski, Karolina Kłoda, Andrzej Pawlik, Krzysztof Safranow, Kazimierz Ciechanowski

**Affiliations:** 1 Clinical Department of Nephrology, Transplantology and Internal Medicine, Pomeranian Medical University, Szczecin, Poland; 2 Department of Pharmacology, Pomeranian Medical University, Powst. Wlkp. 72, 70-111 Szczecin, Poland; 3 Department of Biochemistry and Medical Chemistry, Pomeranian Medical University, Szczecin, Poland

**Keywords:** IL2-IL21, Polymorphism, Graft, Kidney

## Abstract

**Background:**

Cytokines are key mediators of the immune response after transplantation. The interleukin (IL)-2 cytokine family, which includes IL-2, IL-4, IL-7, IL-9, IL-15, and IL-21, is of particular interest because of its importance in the allogenic response. The aim of this study was to examine the association between the rs6822844 gene polymorphism in the *IL2*-*IL21* region and allograft function after kidney transplantation.

**Methods:**

The study enrolled 270 Caucasian kidney allograft recipients (166 males and 104 females, mean age 47.63 ± 12.96 years). Following parameters were recorded in each case: recipient’s age, delayed graft function (DGF), occurrence and number of episodes of acute rejection (AR), and chronic allograft dysfunction (CAD). Genotyping of the rs6822844 *IL2*-*IL21* cluster gene polymorphism was performed using real-time PCR assay.

**Results:**

There were no statistically significant differences in the genotypes and alleles of the rs6822844 *IL2*-*IL21* cluster gene polymorphism among patients with DGF (*p* = 0.72), AR (*p* = 0.69) and CAD (*p* = 0.77), or in creatinine concentrations 1, 3, 6, 12, 24 or 36 months after transplantation (*p* = 0.46, *p* = 0.58, *p* = 0.6, *p* = 0.72, *p* = 0.7, *p* = 0.76, respectively).

**Conclusion:**

It seems that the rs6822844 *IL2*-*IL21* gene cluster polymorphism is of little importance in allograft function after kidney transplantation.

## Introduction

Cytokines are key mediators of the immune response after transplantation [1]. Particular attention has been paid to the interleukin (IL)-2 cytokine family, which includes IL-2, IL-4, IL-7, IL-9, IL-15, and IL-21, because of their importance in the allogenic response [2]. Structural resemblance and the common γ-chain of their receptors result in functional similarities between these family members [3]. IL-2, which is produced by T cells during an immune response, is crucial for their growth, proliferation, differentiation, and death. It is also involved in T-cell functions such as cytotoxicity and immune regulation [4]. The secretion of IL-2 and the expression of IL-2 receptors (IL-2R) are stimulated by the antigen binding to the T-cell receptor (TCR) [5].

The recently described IL-21 is of great immunological interest. This cytokine is expressed in activated human CD4^+^ T cells and in NK cells, thereby regulating their function [6]. Biological effects of the IL-2 cytokine family are mediated through the γ-chain of the receptor, thus initializing the Jak/STAT pathway and utilizing Jak1, Jak3, and a STAT3 homodimer to activate the target genes [7]. Hence, there is functional overlap between IL-2 and IL-21, including co-stimulation of T-cell proliferation and NK-cell expansion [8]. It has been shown that IL-21 is critical for the autoimmune response and graft-versus-host disease (GVHD) [9–11]. Moreover, expression of IL-21R is upregulated in acute renal allograft rejection [12].

The rate of cytokine synthesis has a genetic influence [13]. There are gene polymorphisms that can influence the expression of IL-2 and IL-21 and may therefore affect the immune response and allograft function after kidney transplantation. Human *IL21* gene is about 8.43 kb and maps to chromosome 4, 180 kb from the *IL2* gene, which means that IL-2 and IL-21 are encoded by the *IL2*-*IL21* gene cluster. A single nucleotide rs6822844 polymorphism has been identified within the *IL2*-*IL21* gene cluster and linked with a number of autoimmune diseases, including type 1 diabetes, ulcerative colitis, Crohn’s disease, celiac disease, systematic lupus erythematosus, and rheumatoid arthritis [14]. However, the role of this polymorphism in solid organ transplantation has not yet been established.

The aim of this study was to examine the impact of the rs6822844 gene polymorphism in the *IL2*-*IL21* region on immune response and allograft function after kidney transplantation.

## Materials and methods

The study enrolled 270 Caucasian kidney allograft recipients (166 males and 104 females, mean age 47.63 ± 12.96 years; transplantation performed between 2000 and 2006). The subjects were selected consecutively from among deceased donor renal transplant recipients. Individuals in possession of a functioning graft for <6 months were excluded from the study. Patients were observed in the Clinical Department of Nephrology, Transplantology and Internal Medicine of the Pomeranian Medical University in Szczecin, Poland. The causes of renal failure were: chronic glomerulonephritis (58 %), hypertension (9 %), diabetes (9 %), chronic pyelonephritis (5 %), systemic lupus erythematosus (4 %), autosomal dominant polycystic kidney disease (4 %), vesicoureteral reflux (3 %), and Alport syndrome (1 %). The cause of renal failure remained unknown in 7 % of the studied patients. Hemodialysis was applied to 94 % of the subjects, 3 % were undergoing peritoneal dialysis and 3 % qualified for preemptive kidney transplantation. Comorbid conditions were: arterial hypertension, type 1 and 2 diabetes, atherosclerosis, cardiovascular disease, autoimmune disorders, osteoarthritis, and osteoporosis. The mean number of matching HLA-A and HLA-B alleles was 1.5 ± 0.8 (out of four possible), and the mean number of matching HLA-DR alleles was 1.2 ± 0.5 (out of two possible). Following parameters were recorded in each case: recipient’s age and gender, delayed graft function (DGF), which was defined as the need for dialysis in the first 7 days after transplantation, occurrence and number of episodes of acute rejection (AR), and chronic allograft dysfunction (CAD). CAD was defined by identification of interstitial fibrosis and tubular atrophy on allograft biopsy samples [15]. Blood samples were collected from all patients for genetic analysis at the start of the study and for the evaluation of creatinine concentration 1, 3, 4, 12, 24, and 36 months after kidney transplantation. Creatinine concentration was measured using a colorimetric method. Deteriorating renal transplant function was verified via biopsy, which was reviewed by a renal pathologist using the Banff working classification criteria. In the case of AR, the presence of antibody-mediated rejection only was confirmed in one patient. Other cases of AR were cell-mediated rejection and mixed type.

All AR episodes were proven on allograft biopsy samples. All patients received a standard immunosuppressive protocol with triple drug therapy including calcineurin inhibitor (cyclosporine A in 75 %, or tacrolimus in 24 % of recipients), azathioprine (55 %) or mycophenolate mofetil (37 %), and steroids (91 %). Informed consent was obtained from all patients. Local ethics committee of the Pomeranian Medical University in Szczecin, Poland, approved the study protocol.

### Genotyping

SNP within the *IL2*-*IL21* gene (rs6822844) was genotyped using TaqMan genotyping assays. Genomic DNA was extracted from 200 µL of whole-blood samples using a GeneMATRIX Quick Blood DNA Purification Kit (EURx, Poland). Fluorescence data were captured using a 7500 FAST Real-Time PCR System (Applied Biosystems, USA).

### Statistical analysis

The distribution of the genotypes and alleles was evaluated using the Chi-square test with Yates’ correction or Fishers’ exact test. Serum concentrations of creatinine were compared between genotype groups using the nonparametric Kruskal–Wallis test followed by the Mann–Whitney test. A *p* value <0.05 was considered statistically significant.

## Results

Delayed graft function was diagnosed in 85 (31.5 %) individuals. Comparison of the distribution of genotypes and alleles of the rs6822844 *IL2*-*IL21* cluster gene polymorphism among patients with and without DGF showed no statistically significant differences (*p* = 0.72) (Table 1).

**Table 1:** *IL2*-*IL21* gene cluster polymorphism in patients with and without delayed graft function

	DGF		Without DGF		*p* value^^^		*p* value*	OR (95 % CI)
	*n*	%	*n*	%				
*IL2/IL21* rs6822844:G>T genotype								
GG	67	32.06	142	67.94	0.72	TT+GT versus GG	0.67	0.89 (0.48–1.65)
GT	17	30.91	38	69.09		TT versus GT+GG	0.67	0.43 (0.05–3.73)
TT	1	16.67	5	83.33		TT versus GG	0.67	0.42 (0.05–3.70)
						GT versus GG	1.00	0.95 (0.50–1.80)
						TT versus GT	0.66	0.45 (0.05–4.12)
*IL2/IL21* rs6822844:G>T allele								
G	151	88.82	322	87.03				
T	19	11.18	48	12.97		T versus G	0.67	0.84 (0.48–1.49)

^ Test *χ* ^2^

* Fisher’s exact test

Likewise, the distribution of genotypes and alleles of the rs6822844 *IL2*-*IL21* cluster gene polymorphism among patients with and without AR did not differ significantly (*p* = 0.69) (Table 2).

**Table 2:** *IL2*-*IL21* gene cluster polymorphism in patients with and without acute rejection

	Acute rejection		Without acute rejection		*p* value^^^		*p* value*	OR (95 % CI)
	*n*	%	*n*	%				
*IL2/IL21* rs6822844:G>T genotype								
GG	56	26.79	153	73.21	0.69	TT+GT versus GG	0.62	0.81 (0.42–1.59)
GT	12	21.82	43	78.18		TT versus GT+GG	0.65	1.44 (0.26–8.05)
TT	2	33.33	4	66.67		TT versus GG	0.66	1.37 (0.24–7.67)
						GT versus GG	0.49	0.76 (0.38–1.55)
						TT versus GT	0.61	1.79 (0.29–10.99)
*IL2/IL21* rs6822844:G>T allele								
G	124	88.57	349	87.25				
T	16	11.43	51	12.75		T versus G	0.77	0.88 (0.49–1.61)

^ Test *χ* ^2^

* Fisher’s exact test

CAD was diagnosed in 62 (23 %) individuals. Analysis of the distribution of the genotypes and alleles of the rs6822844 *IL2*-*IL21* cluster gene polymorphism in patients with and without CAD is presented in Table 3. None of the differences were statistically significant.

**Table 3:** *IL2*-*IL21* gene cluster polymorphism in patients with and without chronic allograft dysfunction

	Chronic allograft dysfunction		Without chronic allograft dysfunction		*p* value^^^		*p* value*	OR (95 % CI)
	*n*	%	*n*	%				
*IL2/IL21* rs6822844:G>T genotype								
GG	50	23.92	159	76.08	0.77	TT+GT versus GG	0.60	0.78 (0.38–1.58)
GT	11	20.00	44	80.00		TT versus GT+GG	1.00	0.67 (0.08–5.81)
TT	1	16.67	5	83.33		TT versus GG	1.00	0.64 (0.07–5.57)
						GT versus GG	0.59	0.80 (0.38–1.65)
						TT versus GT	1.00	0.80 (0.08–7.56)
*IL2/IL21* rs6822844:G>T allele								
G	111	89.52	362	87.02				
T	13	10.48	54	12.98		T versus G	0.54	0.79 (0.41–1.49)

^ Test *χ* ^2^

* Fisher’s exact test

Creatinine concentrations 1, 3, 6, 12, 24, and 36 months after transplantation did not differ significantly between the different genotypes and alleles of the rs6822844 *IL2*-*IL21* cluster gene polymorphism (Table 4). Likewise, the age of the recipient and the number of AR episodes were not associated with the rs6822844 *IL2*-*IL21* cluster gene polymorphism (*p* = 0.97 and *p* = 0.67, respectively) (Table 5).

**Table 4:** Associations between creatinine concentrations and the genotypes of the rs6822844 *IL2*-*IL21* cluster gene polymorphism

Creatinine (mg/dl)	*IL2/IL21* rs6822844:G>T genotype						*p* value^^^	GG versus GT
	GG		GT		TT			*p* value*
	*n*	Mean ± SD	*n*	Mean ± SD	*n*	Mean ± SD		
1 month	209	1.86 ± 0.94	55	1.81 ± 0.57	6	2.09 ± 0.98	0.46	0.32
3 months	209	1.76 ± 0.62	55	1.72 ± 0.55	6	2.00 ± 0.73	0.58	0.75
6 months	208	1.77 ± 0.67	55	1.80 ± 0.52	6	1.91 ± 0.73	0.60	0.38
12 months	204	1.74 ± 0.59	54	1.83 ± 0.68	6	1.81 ± 0.64	0.72	0.50
24 months	200	1.76 ± 0.62	50	1.66 ± 0.48	6	1.86 ± 0.85	0.70	0.46
36 months	188	1.73 ± 0.60	46	1.68 ± 0.54	6	2.02 ± 1.13	0.76	0.74

^^^Kruskal–Wallis test

* Mann–Whitney test

**Table 5:** Associations between recipient’s age, acute rejection episodes, and the genotypes of the rs6822844 *IL2*-*IL21* cluster gene polymorphism

	*IL2/IL21* rs6822844:G>T genotype						*p* value^^^	GG versus GT
	GG		GT		TT			*p* value*
	*n*	Mean ± SD	*n*	Mean ± SD	*n*	Mean ± SD		
Recipient’s age	209	47.49 ± 12.96	55	47.98 ± 13.25	6	49.00 ± 11.90	0.97	0.91
AR episode	209	0.31 ± 0.56	55	0.27 ± 0.59	6	0.50 ± 0.84	0.67	0.49

^^^Kruskal–Wallis test

* Mann–Whitney test

## Discussion

We found no statistically significant association between the rs6822844 gene polymorphism in the *IL2*-*IL21* region and DGF, AR, CAD, or creatinine concentration after kidney transplantation. There was also no association between the studied polymorphism and the recipients’ age and number of AR episodes.

In earlier studies, we demonstrated that *ICAM1, VCAM1, CTLA4*, and *PTPN22* gene polymorphisms affect the immune response and kidney function after transplantation. We indicated that the rs5498 *ICAM1* gene polymorphism is associated with risk of AR occurrence, grade of interstitial fibrosis in renal allograft biopsy, and long-term organ function [16, 17]. The rs1041163 *VCAM1* gene may affect long-term allograft failure, graft loss, and overall mortality, and rs231775 *CTLA4* gene polymorphism may be associated with risk of DGF [18, 19]. These findings led us to hypothesize that functional polymorphisms within interleukin genes might be linked to immune response and allograft function after transplantation. We chose the rs6822844 *IL2*-*IL21* gene cluster polymorphism because of its promising character in studies on autoimmune diseases and the involvement of IL-2 and IL-21 in the rejection process.

Acute rejection after kidney transplantation occurs mainly in the first year after transplantation, with the highest risk during the first 6 months. The most frequent form is acute cellular rejection, which is diagnosed by kidney biopsy, showing tubulointerstitial CD4 and CD8 lymphocyte infiltration of the allograft and negative C4d staining [20]. Antibody-mediated rejection or acute humoral rejection is less frequent, and on pathological examination, the presence of leukocytes and positive C4d staining can be seen [21]. In vivo, both types of AR can occur separately or together with different severity. During the cell-mediated rejection process, type 1 helper T-cells and CD8 lymphocytes secrete interferon-γ and IL-2 on alloantigen contact, resulting in activation of cytotoxic T lymphocytes, NK cells, and monocytes. IL-2 is a key mediator of the cellular response [2, 12]. Pathological examination performed in our study revealed cell-mediated rejection separately or coexisting with humoral rejection in almost all AR cases confirmed by biopsy. Therefore, we did not distinguish different subgroups of AR patients in the analysis.

In vitro studies demonstrated that allostimulated T-cells showed a tenfold induction of IL-21 and IL-21R. Higher expression of IL-21 was dependent on IL-2. This was confirmed through inhibition of IL-21 transcription in the presence of anti-IL-2, anti-IL-2R, and immunosuppressive agents. Therefore, it seems that IL-21 is crucial for the IL-2-dependent immune response [1]. Animal GVHD models have also confirmed the increased expression of IL-21 in male and female mice during the immune response. The disease was more severe in females with an almost 55-fold elevation in IL-21 expression [22]. Another study on mice regarding GVHD showed that abrogation of donor T-cell IL-21 signaling resulted in reduced disease severity [23].

Alterations in IL-21 expression have been observed not only in autoimmune and GVH diseases, but also in a rat model of acute renal allograft rejection. Hecker et al. transplanted kidneys between different rat strains and isolated intravascular, spleen, and thymus leukocytes from the recipients 4 days after transplantation. They found that IL-21R was upregulated by the mononuclear leukocytes accumulating inside blood vessels of the kidney allograft during AR. Expression of IL-21 was also higher because of CD4^+^ cell activation during the course of the immune response [12]. This cytokine is considered important in acute allograft rejection. The pro-inflammatory action of IL-21 on CD8^+^ cells leads to their proliferation and cytotoxic state acquisition and subsequent graft destruction. Similarly, IL-21 initiates increased generation of NK cells and promotes their maturation. Strong upregulation of IL-21 and IL-21R via IL-2 has also been observed in biopsies of human heart allografts during AR [2]. Considering the role of IL-21 in transplantation gave new insights into the cytoprotective role of immunoregulatory T-cells (Tregs) and therefore the induction of allograft tolerance [24]. It has been shown that IL-21 has a potential inhibitory influence on Tregs through their dysfunction or induction of apoptosis. Breaking the tolerance leads to an enhanced alloimmune response. The blockade of IL-21/IL-21R could be beneficial in preventing AR episodes [25].

The negative results obtained in our study are not comparable to those of earlier studies, because we did not analyze IL-2 and IL-21 expression. The other limitations of our study are that since the study was conducted only on Caucasian population, its results cannot be generalized to other races. In addition, the results of this study are only applicable to cadaveric donor transplant population with graft survivals of more than 6 months, and the results could have been modified if all patients were included. The functional character of the rs6822844 *IL2*-*IL21* gene cluster polymorphism has been confirmed in numerous studies of autoimmune diseases. There are no reports on this polymorphism in solid organ transplantation. We found no correlation between the rs6822844 *IL2*-*IL21* gene cluster polymorphism and DGF, AR, CAD, and allograft function after kidney transplantation.

## Conclusion

We conclude that there is no association of rs6822844 IL2-IL21 gene cluster polymorphisms with important determinants of graft function in Caucasian population receiving cadaveric donor transplants.

## References

[1] Sadeghi M, Daniel V, Weimer R (2003). Differential early posttransplant cytokine responses in living and cadaver donor renal allografts. Transplantation.

[2] Baan CC, Balk AH, Dijke IE, Korevaar SS, Peeters AM, de Kuiper RP (2007). Interleukin-21: an interleukin-2 dependent player in rejection processes. Transplantation.

[3] Di Carlo E, Comes A, Orengo AM, Rosso O, Meazza R, Musiani P (2004). IL-21 induces tumor rejection by specific CTL and IFN-gamma-dependent CXC chemokines in syngeneic mice. J Immunol.

[4] Millán O, Rafael-Valdivia L, Torrademé E, López A, Fortuna V, Sánchez-Cabus S (2013). Intracellular IFN-γ and IL-2 expression monitoring as surrogate markers of the risk of acute rejection and personal drug response in de novo liver transplant recipients. Cytokine.

[5] Satake A, Schmidt AM, Archambault A, Leichner TM, Wu GF, Kambayashi T (2013). Differential targeting of IL-2 and T cell receptor signaling pathways selectively expands regulatory T cells while inhibiting conventional T cells. J Autoimmun.

[6] Chen YH, Kuo ML, Cheng PJ, Hsaio HS, Lee PT, Lin SJ (2012). Regulation of CD28 expression on umbilical cord blood and adult peripheral blood CD8+ T cells by interleukin(IL)-15/IL-21. Cytokine.

[7] Habib T, Senadheera S, Weinberg K, Kaushansky K (2002). The common gamma chain (gamma c) is a required signaling component of the IL-21 receptor and supports IL-21-induced cell proliferation via JAK3. Biochemistry.

[8] Takaki R, Hayakawa Y, Nelson A, Sivakumar PV, Hughes S, Smyth MJ (2005). IL-21 enhances tumor rejection through a NKG2D-dependent mechanism. J Immunol.

[9] McGuire HM, Walters S, Vogelzang A, Lee CM, Webster KE, Sprent J (2011). Interleukin-21 is critically required in autoimmune and allogeneic responses to islet tissue in murine models. Diabetes.

[10] Meguro A, Ozaki K, Oh I, Hatanaka K, Matsu H, Tatara R (2010). IL-21 is critical for GVHD in a mouse model. Bone Marrow Transpl.

[11] Oh I, Ozaki K, Meguro A, Hatanaka K, Kadowaki M, Matsu H (2010). Altered effector CD4+ T cell function in IL-21R-/- CD4+ T cell-mediated graft-versus-host disease. J Immunol.

[12] Hecker A, Kaufmann A, Hecker M, Padberg W, Grau V (2009). Expression of interleukin-21, interleukin-21 receptor alpha and related type I cytokines by intravascular graft leukocytes during acute renal allograft rejection. Immunobiology.

[13] Akalin E, Murphy B (2001). Gene polymorphisms and transplantation. Curr Opin Immunol.

[14] Hollis-Moffatt JE, Chen-Xu M, Topless R, Dalbeth N, Gow PJ, Harrison AA (2010). Only one independent genetic association with rheumatoid arthritis within the KIAA1109-TENR-IL2-IL21 locus in Caucasian sample sets: confirmation of association of rs6822844 with rheumatoid arthritis at a genome-wide level of significance. Arthritis Res Ther.

[15] Chapman JR, O’Connell PJ, Nankivell BJ (2005). Chronic renal allograft dysfunction. J Am Soc Nephrol.

[16] Kłoda K, Domanski L, Pawlik A, Kurzawski M, Safranow K, Ciechanowski K (2010). Effect of the *ICAM1* and *VCAM1* gene polymorphisms on delayed graft function and acute kidney allograft rejection. Ann Transpl.

[17] Domanski L, Kłoda K, Pawlik A, Wisniewska M, Kwiatkowska E, Kurzawski M (2013). Correlation between *ICAM1* and *VCAM1* gene polymorphisms and histopathological changes in kidney allograft biopsies. Arch Med Sci.

[18] Kłoda K, Domański L, Pawlik A, Safranow K, Ciechanowski K (2013). The impact of *ICAM1* and *VCAM1* gene polymorphisms on long-term renal transplant function and recipient outcomes. Ann Transpl.

[19] Kłoda K, Domanski L, Bobrek-Lesiakowska K, Pawlik A, Safranow K (2013). The impact of *CTLA4* and *PTPN22* genes polymorphisms on long-term renal allograft function and transplant outcomes. Ren Fail.

[20] Ganji MR, Broumand B (2007). Acute cellular rejection. Iran J Kidney Dis.

[21] Puttarajappa C, Shapiro R, Tan HP (2012) Antibody-mediated rejection in kidney transplantation: a review. J Transpl 2012:193724 10.1155/2012/193724 PMC3337620 22577514

[22] Foster AD, Haas M, Puliaeva I, Soloviova K, Puliaev R, Via CS (2010). Donor CD8 T cell activation is critical for greater renal disease severity in female chronic graft-vs.-host mice and is associated with increased splenic ICOS(hi) host CD4 T cells and IL-21 expression. Clin Immunol.

[23] Hanash AM, Kappel LW, Yim NL, Nejat RA, Goldberg GL, Smith OM (2011). Abrogation of donor T-cell IL-21 signaling leads to tissue-specific modulation of immunity and separation of GVHD from GVL. Blood.

[24] Franzese O, Mascali A, Capria A, Castagnola V, Paganizza L, Di Daniele N (2013). Regulatory T cells in the immunodiagnosis and outcome of kidney allograft rejection. Clin Dev Immunol.

[25] Petrelli A, Carvello M, Vergani A, Lee KM, Tezza S, Du M (2011). IL-21 is an antitolerogenic cytokine of the late-phase alloimmune response. Diabetes.

